# Impact of Soluble HLA-G Levels and Endometrial NK Cells in Uterine Flushing Samples from Primary and Secondary Unexplained Infertile Women

**DOI:** 10.3390/ijms16035510

**Published:** 2015-03-10

**Authors:** Roberta Rizzo, Giuseppe Lo Monte, Daria Bortolotti, Angela Graziano, Valentina Gentili, Dario Di Luca, Roberto Marci

**Affiliations:** 1 Department of Medical Sciences, Section of Microbiology and Genetics, University of Ferrara, 44100 Ferrara, Italy; E-Mails: roberta.rizzo@unife.it (R.R.); brtdra@unife.it (D.B.); valentina.gentili@unife.it (V.G.); dario.diluca@unife.it (D.D.L.); 2 Department of Morphology, Surgery and Experimental Medicine, Section of Gynecology and Obstetrics, University of Ferrara, 44100 Ferrara, Italy; E-Mails: angela.graziano@unife.it (A.G.); roberto.marci@unife.it (R.M.); 3 University Hospital “S. Anna”, 44100 Ferrara, Italy

**Keywords:** primary infertility, secondary infertility, soluble human leukocyte antigen (sHLA-G), uterine flushing, endometrial natural killer, CD158d (KIR2DL4)

## Abstract

The aim of this research was to determine the levels of human leukocyte antigen G (HLA-G) and endometrial Natural Killer ((e)NK) cell percentages in uterine flushing samples from primary and secondary infertile women. sHLA-G levels were lower in the uterine flushing samples from primary infertile women in comparison with women with secondary infertility. Lower CD56^+^KIR2DL4^+^ (e)NK cell percentages were detected in primary infertile women compared with secondary infertile women. This is the first study demonstrating that primary and secondary unexplained infertilities are characterized by different basal sHLA-G levels and CD56^+^KIR2DL4^+^ (e)NK cell percentages.

## 1. Introduction

A complex, highly coordinated sequence of structural and biochemical changes culminate in the generation of a “window” of uterine receptivity during the mid-luteal phase of each menstrual cycle. Compromised receptivity of the endometrium is believed to be a primary cause of unexplained infertility characterized by implantation failure and subclinical pregnancy loss. In women, unexplained infertility has been associated with a range of cellular and molecular defects of the endometrium and immunological factors. Since the fetus is semi-allogenic respect to the mother, the maternal immune-modulation is crucial in protecting the fetus.

Natural Killer (NK) cells are the dominant immune cell type in the endometrium and play a major role in determining pregnancy outcome [1]. Human decidual (d)NK cells are a distinct CD56^bright^CD16^−^ NK cell subset with a reduced cytotoxicity. They express specific markers such as CD9 and CD49a [2] and inhibitory receptors (*i.e.*, KIR2DL4, LILRB1 and LILRB2), known to interact with Human Leukocyte Antigen (HLA)-G molecules [3]. HLA-G antigens are non classical HLA class I molecules characterized by seven isoforms obtained by mRNA alternative splicing: four membrane-bound (HLA-G1–G4) and three soluble isoforms (HLA-G5–G7) [4] and a tolerogenic function during pregnancy. In particular lower levels of sHLA-G in maternal blood were associated with pregnancy complications as pre-eclampsia and recurrent pregnancy loss [4]. Moreover, HLA-G expression is important also during oocytes maturation and embryos implantation [5]. HLA-G molecules are expressed by cytotrophoblast cells at the maternal-fetus interface where they bind KIR2DL4 receptor expressed by the uterine NK cells [3]. This interaction controls the activation of the uterine NK cells and promotes the formation of the placenta [1]. Interestingly, sHLA-G was detected both in testis and in seminal fluid with significant differences among fertile and infertile couples [6]. Moreover, the decrease in CD56^bright^CD16^−^ dNK cells and HLA-G expression have been associated with recurrent miscarriage, suggesting their relevance in pregnancy outcome [7]. (d)NK cells have been extensively investigated during pregnancy, while endometrial (e)NK cells still lacks comprehensive researches.

We evaluated the role of sHLA-G in uterine flushing from women affected by primary or secondary infertility and the possible correlation with (e)NK presence.

## 2. Results

**Figure 1:**
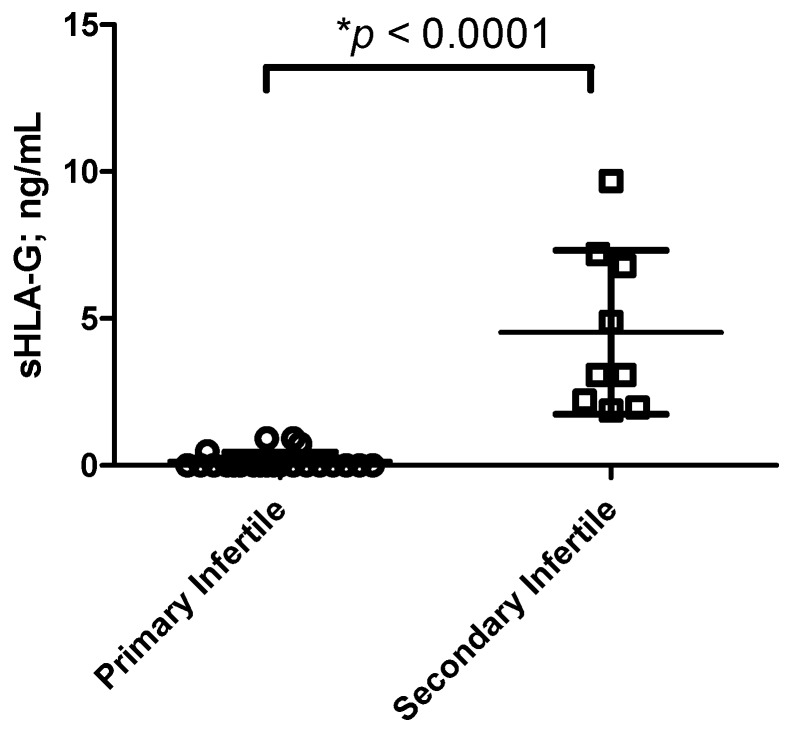
sHLA-G levels (mean ± SD) in primary and secondary infertility. sHLA-G levels were normalized for flushing volume and total protein content. * Student *t* test.

**Figure 2:**
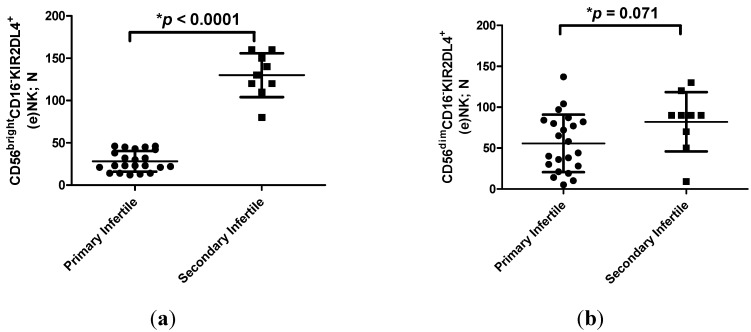
(**a**) Number of CD56^bright^CD16^−^KIR2DL4^+^ (e)NK cells (mean ± SD) in flushing samples from primary and secondary infertile women. * Student *t* test; (**b**) Number of CD56^dim^CD16^−^KIR2DL4^+^ (e)NK cells (mean ± SD) in flushing samples from primary and secondary infertile women. * Student *t* test.

All patients were characterized by years of sterility (Table 1). Hormonal and demographic parameters (Follicle-stimulating hormone (FSH), Luteinizing hormone (LH), 17-β-Estradiol, progesterone, prolactin, Thyroid-stimulating hormone (TSH) and free thyroxine (FT4) levels, smoke habits, age and weight) proved not to be related with primary and secondary infertility (Table 1). The only difference was in TSH levels, with lower values in secondary infertile women (*p =* 0.0041; Student *t* test). However, the TSH concentrations ranged between the normal values (0.27–4.20 µUI/mL) in both the group of women, excluding any pathological condition. Moreover, we observed no correlation between TSH levels and the condition of primary or secondary infertility (*r* = −0.212; *p =* 0.252; Spearman correlation test). The evaluation of sHLA-G levels in the uterine flushing samples from primary and secondary infertile women revealed higher levels of sHLA-G in secondary infertile women (mean ± standard deviation: 4.53 ± 2.78 ng/mL) compared with primary infertile women (0.14 ± 0.31 ng/mL) (*p* < 0.0001; Student *t* test) (Figure 1). Interesting, we observed a significant correlation between sHLA-G levels in uterine flushing samples and the condition of primary or secondary infertility (*r* = 0.80; *p =* 7.3 × 10^−8^). The analysis of cell content in uterine flushing samples (Table 2) showed a lower amount of (e)NK cells in the uterine flushing samples of primary infertile women compared with secondary infertile women (*p* < 0.0001; Student *t* test) (Table 2). In particular, when we analyzed (e)NK cell subpopulations, we observed a lower percentage of CD56^bright^CD16^−^KIR2DL4^+^ (e)NK cells in the uterine flushing samples of primary infertile women compared with secondary infertile women (*p* < 0.0001; Student *t* test) (Figure 2a). On the contrary, no differences were observed between CD56^dim^CD16^−^KIR2DL4^+^ (e)NK cells in the two groups of women (*p =* 0.071; *t* test) (Figure 2b). The uterine flushing samples presented no CD3^+^ cells and a low percentage of CD14^+^ cells (Table 2), with no significant differences between primary infertile and secondary infertile women (*p =* 0.74; Student *t* test) (Table 2).

**Table 1:** Hormonal and demographic parameters of patients.

Items	Primary Infertility	Secondary Infertility	*p* Value
Age	34.7 ± 3.5	35.6 ± 3.4	0.53 *
Duration of Infertility (years)	2.7 ± 2.0	3.1 ± 2.3	0.61 *
Length of menstrual cycle (days)	29.0 ± 4.0	28.2 ± 3.3	0.58 *
FSH (mUI/mL) day 3 of the menstrual cycle	7.6 ± 2.7	6.9 ± 2.9	0.59 *
LH (mUI/mL) day 3 of the menstrual cycle	6.9 ± 3.4	5.0 ± 2.2	0.13 *
Estradiol (pg/mL) day 3 of the menstrual cycle	73.8 ± 65.5	54.9 ± 42.8	0.43 *
TSH (µUI/mL)	3.8 ± 4.1	2.1 ± 1.4	0.0041 *
FT4 (pg/mL)	2.8 ± 3.9	2.2 ± 3.0	0.45
Progesterone (pg/mL) day 21 of the menstrual cycle	13.7 ± 9.6	12.4 ± 2.7	0.76 *
Smoke habits (percentage)	22.7%	11.1%	0.074 **
Day (menstrual cycle) of sample collection	8.9 ± 1.9	9.1 ± 1.8	0.84 *

* Student *t* test; ** Fisher exact test; Follicle-stimulating hormone (FSH); Luteinizing hormone (LH); Thyroid-stimulating hormone (TSH), free thyroxine (FT4).

**Table 2:** Cell count in uterine flushing samples.

Items	Primary Infertility	Secondary Infertility	*p* Value
NK cells *N* (%)	84.1 ± 42.1 (21)	212.1 ± 48.5 (21)	<0.0001 *
CD56^+^CD16^−^ KIR2DL4^+^ *N* (%)	80.1 ± 20.3 (8.4)	200.0 ± 40.1 (20)	<0.0001 *
CD56^bright^CD16^−^KIR2DL4^+^ *N* (%)	28.3 ± 12.1 (2.8)	118.8 ± 2.3 (12)	<0.0001 *
CD56^dim^CD16^−^KIR2DL4^+^ *N* (%)	55.8 ± 35.2 (5.6)	82.1 ± 36.2 (8.2)	0.071 *
CD3^+^ *N* (%)	0	0	NA *
CD14^+^ *N* (%)	3.4 ± 7.9 (0.3)	2.4 ± 4.9 (0.2)	0.74 *

* Student *t* test; Not applicable (NA).

## 3. Discussion

The data obtained in this study support the hypothesis of an implication of sHLA-G molecules in infertility condition. We found different profiles of sHLA-G expression between primary and secondary infertile women. In particular, primary infertile women presented lower levels of sHLA-G in uterine flushing samples if compared with secondary infertile women. Several evidences support the major role of HLA-G in the physiopathology of infertility and the evaluation of sHLA-G expression was suggested as a future tool to select single embryos for transfer in order to reduce the risk of multiple pregnancy and to increase clinical pregnancy outcomes during *in vitro* fertilization protocols [5,8]. Recently, HLA-G was evaluated also in male reproductive system: sHLA-G has been detected in testis and also in semen and semen plasma with significant differences among different men [6] suggesting that a favorable environment for pregnancy could be induced by the presence of sHLA-G molecules in the female reproductive tract that could be also carried by semen after coitus.

Moreover, the analysis of the amount of (e)NK cells showed a lower percentage of CD56^bright^CD16^−^KIR2DL4^+^ (e)NK cells in primary infertile women. It is known that HLA-G antigens act as immune-inhibitory molecules interacting with immune-inhibitory receptors (ILT2, ILT4 and KIR2DL4). During pregnancy, HLA-G molecules interact with (d)NK cells inducing a polarization towards cytokine production [9] and sustaining a correct placentation and embryo implantation. For this, the observation of a different characterization of primary and secondary unexplained infertility with regard to sHLA-G levels and CD56^bright^CD16^−^KIR2DL4^+^ (e)NK cell percentages in uterine flushing samples is of extreme interest. These differences sustain different mechanisms at the basis of these two infertile conditions. Our results suggest that lower sHLA-G levels could prevent the creation of an appropriate tolerogenic uterine environment and a consequent lower presence of CD56^bright^CD16^−^KIR2DL4^+^ (e)NK cells in primary infertility. On the contrary, the high levels of sHLA-G and CD56^bright^CD16^−^KIR2DL4^+^ (e)NK cells found in secondary infertile women uterine flushing samples suggest a different cause at the basis of this condition. In fact, secondary infertile women, unlike primary infertile ones, were able to carry out almost one previous pregnancy. It is interesting to note that the modulation in the number of circulating NK cells seems to be a primary event during inflammatory/autoimmune processes rather than a consequence of inflammation and drug administration, playing a fundamental role in the pathogenesis of a number of autoimmune diseases [10] and, on the basis of our results, also in female infertility.

## 4. Experimental Section

### 4.1. Patient Recruitment

The study was carried out in an inpatient setting following the ethical rules of the Azienda Ospedaliero Universitaria Ferrara, Italy. All patients signed an informed consent for the specimen collection. We enrolled 31 women affected by unexplained infertility (primary infertility, *n* = 22; secondary infertility, *n* = 9). Women were diagnosed with “primary infertility” in case they were both unable to conceive and to carry pregnancy to live birth. Otherwise, the failure to conceive following a previous pregnancy (spontaneous/voluntary abortions or childbirths) was referred to as “secondary infertility”. Patients were recruited at admission for tubal patency assessment by Hystero-sono contrast sonography 7–9 days after menstruation. Inclusion criteria for the study group were: 21–38 years old, regular menstrual cycle (24–35 days), body mass index (BMI) ranging between 18 and 26 kg/m^2^, FSH (day 2–3 of the menstrual cycle) <10 mUI/mL, 17-β-Estradiol < 50 pg/mL (day 2–3 of the menstrual cycle), normal karyotype. Women with endometritis, endometriosis, tubal factor, ovulatory dysfunction, anatomical uterine pathologies and recurrent miscarriage were excluded.

### 4.2. Samples Collection

Uterine flushing was performed with a 14-gauge Foley 3-way balloon catheter (Eschmann) inflating an appropriate (5 mL) amount of sterile physiologic saline solution, as previously described [11]. All samples were stored at −20 °C until the analysis.

### 4.3. sHLA-G Analysis by ELISA

sHLA-G quantification in endometrial flushing was performed by ELISA [12] using anti-HLA-G (G233) and anti-β2-microglobulin HRP-conjugated moAbs (Exbio, Praha, Czech). sHLA-G levels were normalized for flushing volume and total protein content ((sHLA-G, ng/mL/total flushing sample, mL)/total proteins, ng/mL). Standard supernatants of HLA-G/721.221 were utilized for the generation of standard calibration curves. The limit of sensitivity was 1 ng/mL.

### 4.4. (e)NK Cell Analysis by Flow Cytometry

(e)NK cells were obtained from flushing sample pellets and were analyzed by flow cytometry with CD3-PerCp, CD14-PE, CD56-PE, CD16-FITC (BD Pharmigen, Erembodegem, Belgium), CD158d-APC (KIR2DL4) (Biolegend, San Diego, CA, USA) monoclonal antibodies.

### 4.5. Statistical Analysis

The data were compared by Student *t* test, as normally distributed according with Kolmogorov-Smirnov test, Fisher exact test and Spearman Correlation test.

## 5. Conclusions

Even though this study is based on a limited number of samples, and no mechanistic data are presented, we report, for the first time, a possible biological difference between primary and secondary unexplained infertility, based on sHLA-G and CD56^bright^CD16^−^KIR2DL4+ (e)NK cells in uterine flushing samples. Further investigations on a larger cohort of subjects, including a control group, different time points during the menstrual cycle, and the evaluation of the biological relationship between HLA-G and (e)NK cells, would be necessary to confirm our data.
